# The ground is the limit: epidemiology of skydiving accidents over 25 years and in 2.1 million jumps in the Netherlands with sub-analysis of injuries reported by medical professionals in the past five years

**DOI:** 10.1186/s13017-024-00535-w

**Published:** 2024-02-28

**Authors:** Michiel Damhuis, Raymond van der Wal, Harriet Frielink, Robert Nijveldt, Joost ten Brinke, Edward Tan

**Affiliations:** 1 Division of Trauma Surgery, Department of Surgery, Radboud University Medical Center, P.O. Box 9101, Nijmegen, 6500 HB The Netherlands; 2 Department of Anesthesiology, Radboud University Medical Center, P.O. Box 9101, Nijmegen, 6500 HB The Netherlands; 3 Division of Trauma Surgery, Department of Surgery, ISALA Hospital, P.O. Box 10400, Zwolle, 8025 AB The Netherlands; 4 Division of Trauma Surgery, Department of Surgery, Gelre Hospital, P.O. Box 9014, Apeldoorn, 7334 DZ The Netherlands

**Keywords:** Emergency medicine, Injuries, Parachute, Prehospital emergency care, Skydiving, Trauma

## Abstract

**Background:**

Skydiving is the fastest nonmotorized sport; and consequently is not without risk. In the last decades, skydiving has become considerably safer but injuries and fatalities still occur. Incidents are reported to and administered by the Royal Netherlands Aeronautical Association (KNVvL). From 1995 to 2020, 2715 incidents were reported; of which 1503 resulted in injury and 26 in fatality. There is a need for more information available on the particular type, severity, and factors which contribute to skydiving-related injuries worldwide. This study aims to investigate patterns in occurrence rates, examine demographic and skydiving-related factors linked to injuries, and analyze the types and severity of injuries relating to these contributing factors.

**Methods:**

The Dutch KNVvL database – covering more than 25 years of data – was examined for contributing factors. An analysis of the severity and types of injury resulting from incidents over the last five years were matched with a search of hospital databases.

**Results:**

The rate of injuries pattern increases starting from 2016, with novice jumpers having the highest risk of injury. Most injuries occur during the landing phase. The lower extremities and the spine are most affected, with fractures being the most prevalent type of injury. More than half of the patients were admitted to hospital, with 10% requiring surgery, resulting in months of rehabilitation.

**Conclusion:**

This study is the first in the Netherlands, and only the second worldwide to analyze *technical* incident databases in combination with data from *medical* information systems. Skydiving accidents of experienced jumpers should be considered as ‘high-energy trauma,’ therefore treatment should follow standard trauma guidelines. In less experienced skydivers, it is critical to conduct a secondary survey to assess the extremities adequately. Clinicians should also pay attention to friction burns that can arise due to friction between the skin and skydive equipment, a phenomenom that is already known in road traffic accidents.

**Supplementary Information:**

The online version contains supplementary material available at 10.1186/s13017-024-00535-w.

## Background

The first successful landing with a parachute from a hot-air balloon was conducted in 1797 by André-Jacques Garnerin. In the twentieth century, militaries further developed the technique of safely reaching the ground by parachute in order to exit a malfunctioning airplane or deliver troops into combat zones. Soon after World War II, parachuting evolved into a popular civilian sport: skydiving. The first world championships were held in 1951. Today, 3,200,000 skydives are conducted annually worldwide, of which approximately 109,150 per year occur in the Netherlands [1, 2].

Skydiving is the fastest nonmotorized sport, and is associated with the risk of severe injury or death. Due to equipment evolution and a developing culture of safety awareness, incidences of injury and fatalities have reduced over the years [3]. Earlier research (from 1985 to 2005) indicates that the injury rate in civilian skydiving ranges from 140 to 174 injuries and 0.85 to 7.7 fatalities per 100,000 jumps [3–6]. In comparison, a recent French study found 49 injuries and 0.57 fatalities per 100,000 jumps [7]. Furthermore, data from the Fédération Aéronautique Internationale (FAI) exhibits the same pattern: 1.14 fatalities per 100,000 jumps in 2009 and 0.64 per 100,000 jumps in 2019 [8, 9].

Despite these decreasing trends, skydiving accidents still occur and can occur during each phase of the jump: on exit, during freefall, during opening, or while suspended under the parachute. However most injuries occur during the landing. Hence, the skydiver’s wisdom: the sky’s not the limit, the ground is [1, 3]. Several prospective or retrospective studies have analyzed skydiving incidents and fatalities [3–7, 10]; with some further analyzing injury type and pattern [11, 12]. Commonly encountered injuries involve fractures occurring in the lower extremities [3, 4, 11]. Risk factors identified in the literature include gender and experience; with less experience the chances of injury increases, while more severe injuries were observed in the more experienced population [7, 11]. A recent large prospective study analyzed skydiving accidents and injuries that were based on self-reported incident databases, rather than by medical professionals [7].

Because of self-reporting, there is a need for more information on the exact nature and severity of skydiving-related injuries, especially in relation to risk factors. Enhancing skydiving safety involves investigation into the frequency and timing of accidents; additionally the individuals involved in these incidents are cruicial to consider. Therefore, the objective of this study is to investigate incident occurrence rate patterns, demographic and skydiving-related factors linked to injuries, and the type and severity of injuries in relation to these contributing factors.

## Methods

### Study design

In this retrospective population study, all skydives were conducted within Dutch skydiving regulations. The minimum age requirement to skydive (solo) is 16 years. The study was conducted with the cooperation of the parachuting section of the Royal Dutch Aeronautical Association (KNVvL; Koninklijke Nederlandse Vereniging voor Luchtvaart). Ethical approval was obtained through the Ethical Review Board of the Radboud University Nijmegen Medical Center (consent number: 202,113,079), the ISALA hospital location Zwolle (consent number: 20,210,912), and the Gelre hospital location Apeldoorn (consent number: 202,169). All data was pseudonymized, coded, and stored at a secured server at the Radboud University Medical Center. Data transfer agreements were established according to Dutch law and internal regulations of the participating hospitals.

### Data collection

In accordance with Article 303 of the ‘basic safety regulations for sports parachuting’ issued by the KNVvL, skydivers in possession of a B license are obligated to report an incident. The instructor on duty must report an incident if an involved sports parachutist does not hold a B license [13]. An incident is defined as any deviation from the normal jump course: (e.g., the deployment of the reserve parachute or landing outside of the intended area), irrespective of whether an injury occurred in combination.

Throughout the years, several reporting data systems have been employed by the KNVvL. The first database concerns incidents until 2009, the second covers incidents from 2010 to 2014, and the last contains incidents from 2015 to 2020. The latter database contains the most detailed information. Due to the incomplete and less detailed data collection from the KNVvL in the first two databases, the exact injury type, experience/currency and type of canopy are unknown for 1,072 cases.

To combine these databases into one dataset, we recoded the variables using a standardized codebook. In this codebook, three categories of variables were identified: type of incident and injury or fatality, jumper demographics, and skydiving-related variables (e.g., the parachute category, meteorological conditions, and experience/currency of the skydiver). Skydivers are defined as current when they have made more than 10 jumps in the preceding 12 months. The data was coded in Microsoft Excel (v. 2018) and transferred to IBM SPSS Statistics (v. 25, SPSS, Inc., Chicago, IL, USA).

To analyze skydiving incidents, we selected a 25 year period, from 1995 (about when a new style of high speed landings using high-performance canopies (called hookturns and swooping) gained popularity, [3] to 2020 (the start of the coronavirus disease (COVID) pandemic).

The complete dataset was analyzed regarding incidents resulting in any physical injury or fatality, and expressed as rates per 100.000 jumps [1]. Patterns of injury related to various mechanisms were identified in the literature and thus categorized in the complete dataset. The patterns included: traumatic brain, (cervical) spine, thoracic, upper extremity, femur, pelvic, and lower extremity injuries or fatalities.

The 2015 to 2020 section of the database was analyzed for contributing factors. First, we related the jump phase to the pattern of injury. To obtain insight into the possible contributing factors, we defined two groups: minor and major injuries. Injuries of the upper or lower arms, wrists/hands, lower legs and ankles/feet were defined as “minor.” Injuries to the other parts of the body, and central plus peripheral injuries are defined as “major,” as described previously in literature [11]. We compared these categories against the jumpers’ demographics (see additional file 1 for more information) and other skydiving-related variables, including meteorological conditions [1].

We concluded the analyses of the last group with specific and detailed medical information from the cases. Injuries in the KNVvL database were self-reported. To analyze specific injuries, cases from the KNVvL database from 2015 to 2020 were matched with hospital data. We narrowed this search to events occuring at the dropzone in Teuge, the largest dropzone in the Netherlands. In 2020, 39% of all Dutch skydives were performed there.

Furthermore, Teuge dropzone has the highest number of jumps per jump type (AOR, AOS, AFF and FF)(see additional file 2 for a detailed description of jump types) in the Netherlands, except for the tandem category. Cases were identified in the medical databases of Level 1 and 2 trauma centers within a 70-km radius of the Teuge drop zone (Radboud University Nijmegen Medical Center, the ISALA hospital location Zwolle, and the Gelre hospital location Apeldoorn). We used CTcue, a program that extracts structured and unstructured patient data from electronic health records (EHRs) to search the system for skydiving incidents with several Dutch medical subject heading terms. The identified cases were matched with the corresponding cases from the KNVvL database by date and other variables, such as gender. Furthermore, the International Severity Score (ISS) was retrieved from the national trauma registry for the included patients.

### Inclusion and exclusion criteria

All registered traumatic skydiving injuries or fatalities in the Netherlands that were reported to the KNVvL from 1995 to 2020 were included. For the subgroup analysis, traumatic skydiving injuries or fatalities at Teuge from 2015 to 2020 which were reported to the KNVvL and can be traced in the medical databases of the participating hospitals, were included. Cases were excluded when data was incomplete or when the injuries were unrelated to the parachute jump.

### Statistical analysis

A statistical analysis was conducted using IBM SPSS (v.25, SPSS, Inc., Chicago, IL, USA). The numeric variables including the jump type, brevet, number of jumps, canopy type and ground wind speed, were compared to the type of injury (minor/major) and described in crosstabs. The Pearson’s chi-square test was applied to assess the statistical significance of the data. An alpha-value less than 0.05 indicated statistical significance.

## Results

### Study population

Between 1995 and 2020, 2,096,866 skydives were carried out in the Netherlands. In this period 2,715 incidents were reported, of which 1,503 resulted in injuries and 26 resulted in fatalities. The median age of all injured skydivers is 26 years (range 16–85). Our entire dataset was examined to determine the relative frequency of incidents leading to injury or fatality [3]. The relative incidence of injury in this study was 72 per 100,000 jumps; and for fatalities, the relative incidence was 1.24 per 100,000 jumps.

To analyze the contributing factors related to skydiving incidents, we used the most complete database (2015–2020), which includes demographic and skydiving-related variables. In this period a total of 514,812 jumps were made in the Netherlands and 1,080 incidents were reported; resulting in 322 injuries and 11 fatalities. Focusing on the largest dropzone in the Netherlands at Teuge, 205.449 skydives were conducted from 2015 to 2020. In addition, 449 incidents were documented, of which 124 resulted in injuries and three in fatalities. Within this data set, 55 patients were identified in the EHRs of three Level 1 and 2 traumacenters within a radius of 70 km from the Teuge dropzone. Furthermore, 30 (24.2% of 124) patients were included in this subgroup analysis.

#### Injury rates, contributing factors and causes

**Fig. 1:**
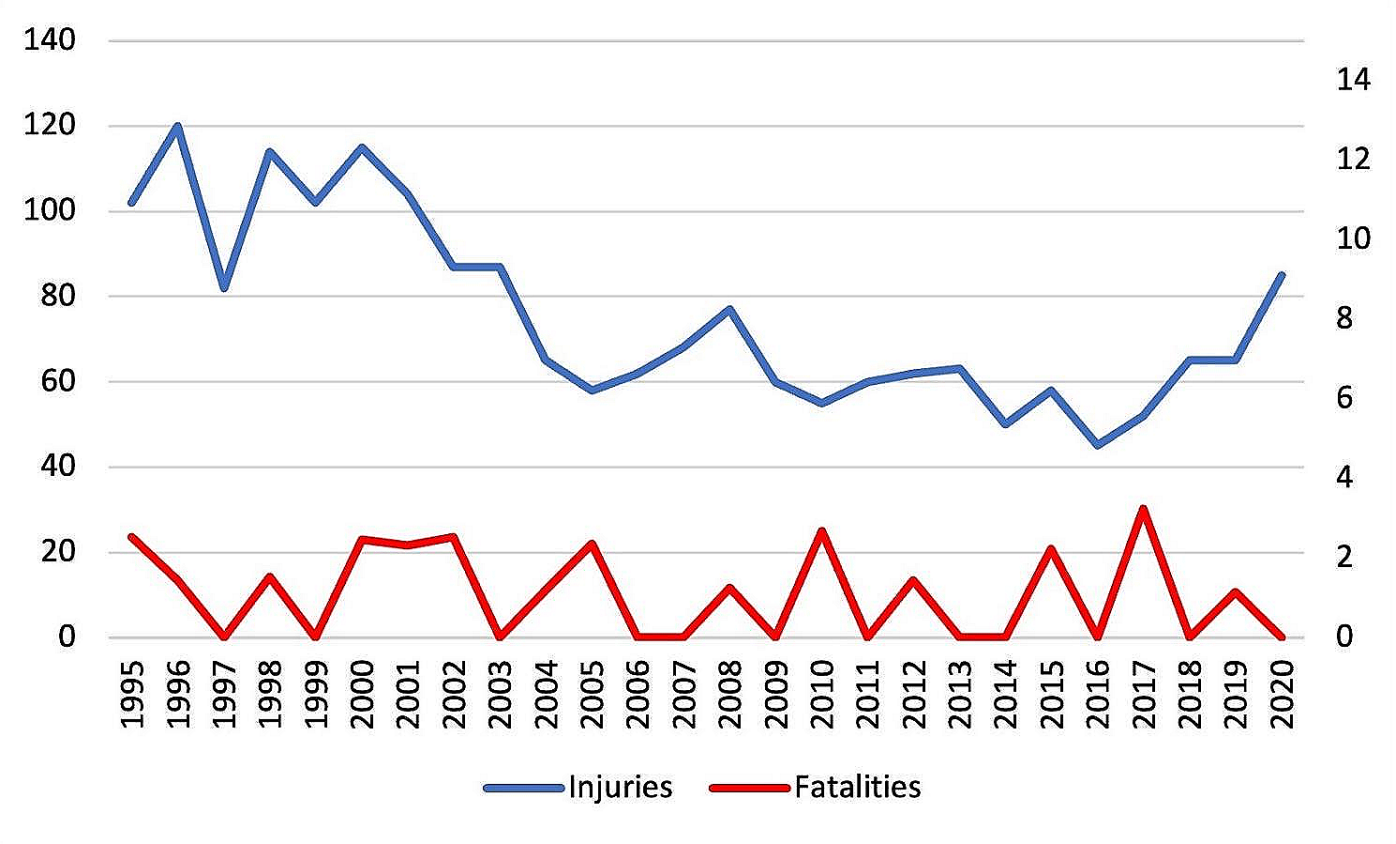
Relative incidence of injuries and fatalities over the years 1995 to 2020. Regarding the incidence of injuries, the data express a downward trend until 2016, after which the trend increases. The incidence of fatalities is relatively stable during the period under study

The overall incidence of fatalities is relatively constant, with zero to three fatalities per 100,000 jumps a year during the 25 years reviewed. Regarding the incidence of injuries, the data express a downward trend until 2016, after which the trend increases (Fig. 1). The more detailed database covering 2015 to 2020 in the Netherlands reveals that highly experienced skydivers with an FF (free fly) jumptype are most likely to experience a major injury. In contrast, inexperienced jumpers, with a more simple AOS (automatic opening square) jump type often suffer minor injuries. This finding reached statistical significance (*p* ≤ 0.001). Moreover meteorological data indicates that skydivers landing at higher ground wind speeds are more likely to sufer a major injury. However no significant difference occurred between these variables (*p* = 0.061). Table 1 lists further details regarding the contributing factors.

**Table 1:** Contributing factors to skydiving related injuries in the period 2015–2020

Contributing factors of all injuries in the period 2015–2020.	Number of skydivers	Unknown	Major injury		Minor injury	Sig.
Injury pattern			Central and peripheral	Central	Peripheral	
Experience/Currency						
Amount of jumps*						P = 0.151
<10	156	10	10	43	93	
10–50	37	4	2	16	15	
50–100	26	0	1	10	15	
100–200	49	4	4	15	26	
>200	30	0	0	17	13	
Unknown	24	3	1	7	13	
Brevet**						P = 0.428
None	183	13	8	58	104	
A	16	0	3	6	7	
B	23	1	1	7	14	
C	7	0	0	3	4	
D	86	4	6	34	42	
Unknown	7	7	0	0	0	
Flight technical						
Jump type**						**P = < 0.001**
AOR	17	2	1	4	10	
AOS	95	8	4	24	59	
AFF	36	2	2	13	19	
FF	126	7	11	44	64	
Tm/p	46	0	0	23	23	
Unknown	2	2	0	0	0	
Canopy type						P = 0.464
Cat I/II	196	12	10	64	110	
Cat III/V	43	2	5	15	21	
Cat VI/VIII	45	1	1	20	23	
Unknown	38	6	2	9	21	
Weather						
Ground wind speed						P = 0.061
<5 m/s	243	14	14	76	139	
5–10 m/s	69	4	4	30	31	
Unknown	10	3	0	2	5	
Wind speed at opening height						N/A
<5 m/s	27	2	4	5	16	
5–10 m/s	108	7	6	34	61	
10–15 m/s	46	5	1	23	17	
15–20 m/s	17	1	0	4	12	
Unknown	124	8	5	42	69	
Cloud cover						N/A
Ceiling and visibility OK	150	10	9	49	82	
Few	53	4	3	20	26	
Scattered	36	0	3	12	21	
Broken	1	0	0	1	0	
Overcast	0	0	0	0	0	
Unknown	82	7	3	26	46	

Legenda. ^*^In last 12 months. ^**^See additional file 1 license, automatic opening round/square, accelerated free fall program and free fall

**Fig. 2:**
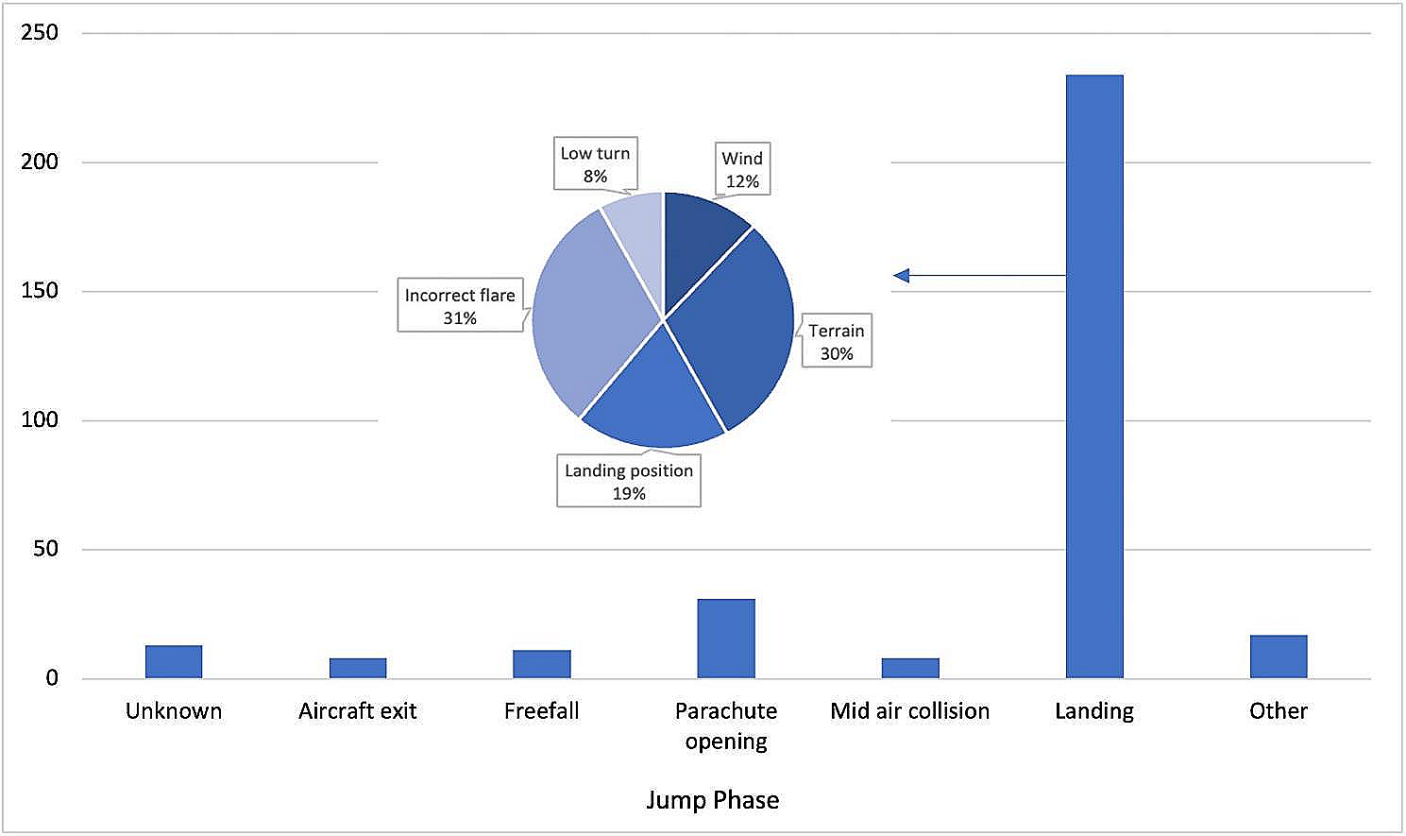
Cause of injury in relation to the jump phase, of all injured skydivers (2015-2020). The figure shows that most injuries occur during the landing phase, with incorrect-flare being the most common cause

The risk of injury during skydiving is the highest during the landing phase of the jump, corresponding to 234 (73%) injuries. An incorrect flare (the technique of slowing a parachute just before landing) (31%), the terrain (30%), and an incorrect landing position (19%) are the most common causes of injury. Of the injured skydivers, 175 (75%) were wearing low sports shoes and 28 (12%) were not wearing a helmet (Fig. 2).

### Anatomical regions, types and ISS of injuries

Regarding the entire sample covering 25 years of skydiving, most injuries were located on the lower extremities (corresponding to 54.8%), versus only 9.5% on the upper extremities and 43.6% on the central part of the body. The most common injuries were fractures, which occurred in 60.2% of the injuries. Contusions and strains accounted for 13.2% and 7.5% of the injuries respectively.

In the most complete database from Teuge in the period 2015 to 2020, the most common injuries for minor injured skydivers were fractures of the lower legs and ankles at six (31.6%) and eight (42.1%) incidents respectively. For the major injured skydivers, the most common injuries were neurological damage of the head, spinal fractures, and pelvis/hip fractures; at four (13%), nine (29%), and three (9.7%) incidents respectively. Half of skydivers who sustained major injury did not have a license (8/19).

**Fig. 3:**
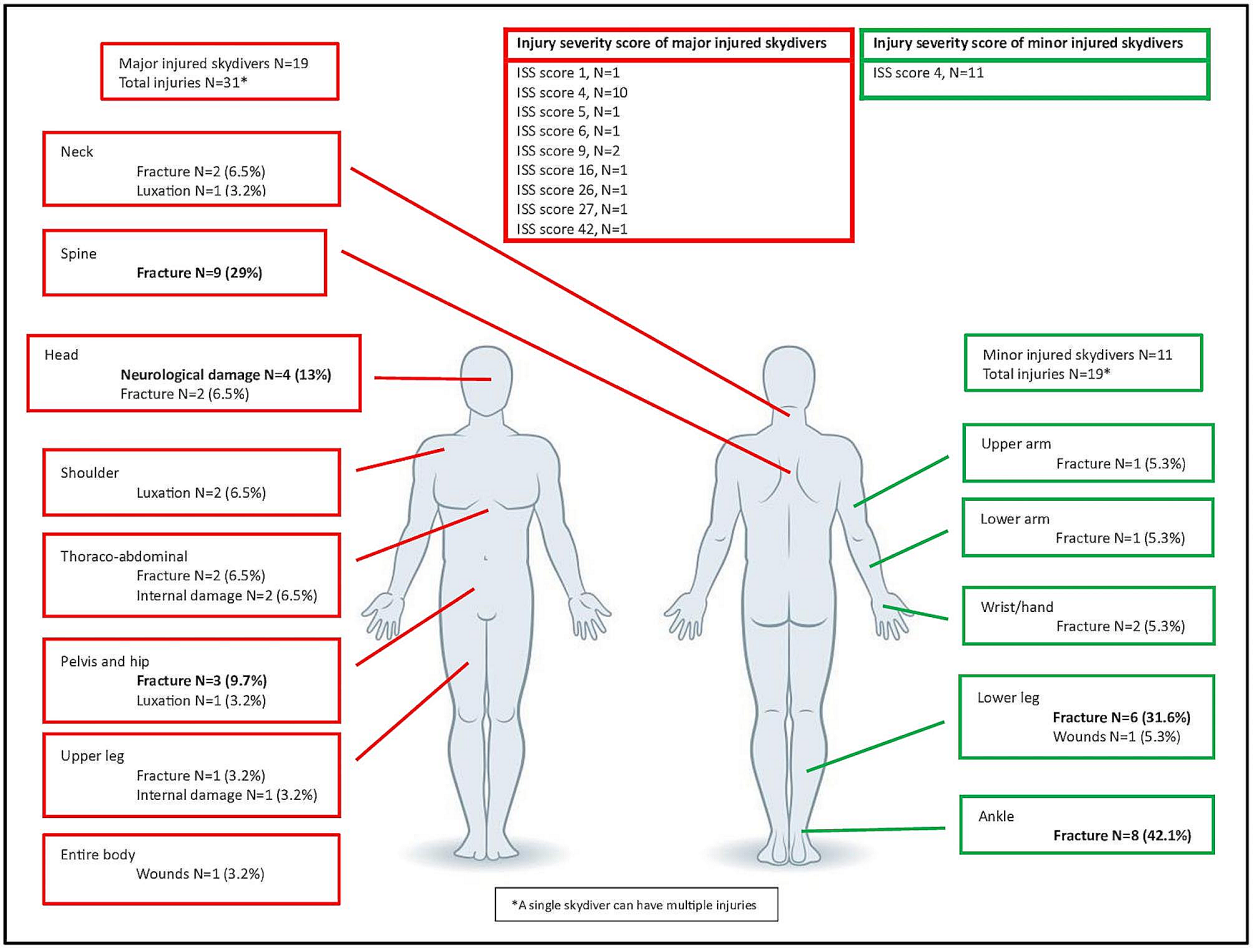
Major and minor injuries. The figure shows detailed information about the type, location, and ISS of major (red) and minor (green) injured skydivers in the period 2015–2020 at Teuge

Using information from the national trauma registry, we calculated the ISS score in the most recent and complete database. The mean ISS of skydivers with minor injuries was 4 (range 0–4), and the mean ISS of skydivers with major injury was 10 (range 1–42). Out of the 18 skydivers who sustained major injury, four had severe injuries with corresponding ISSs of 16, 26, 27, and 42 (Fig. 3).

### Initial care, treatment and rehabilitation of injured skydiers in Teuge 2015–2020

Of the 30 included injured skydivers, nine (30%) patients required a ground ambulance and four (13.3%) patients required helicopter emergency medical services (HEMS) to reach the emergency department. The rest of the injured skydivers arrived by private transport at one of the surrounding hospitals. Compression fractures of the spine, lower leg fractures and bi/tri-malleolus ankle fractures were the most common injuries, observed by clinicians. A quarter of the included patients in this study required surgical treatment, 17 (57%) patients had to be admitted to a hospital for less than a week and five (17%) patients were admitted for more than a week. Ten (33%) patients had to undergo months of rehabilitation due to the injuries they sustained.

## Discussion

To our knowledge, this study is the first in the Netherlands and the most recent worldwide to analyze *technical* incident databases in combination with data from *medical* information systems. The last study dates from 2007 [11]. More recent publications have only analyzed skydiving-related incidents using a technical database [1, 3, 7]. The main findings of this study are that since 2016 the incidence of skydiving accidents has been increasing, whereas the trend was decreasing up to 2016. The data indicates that most injuries occur during the landing phase not only with the inexperienced jumpers, but more importantly with the more experienced skydivers, often resulting in major injuries. Therefore, prevention and protective measures in skydiving should be made mandatory.

### Rise in incidence

Several potential reasons could explain the increase in skydiving injuries documented between 2016 and 2020. For example, the KNVvL stated that the higher number of reports, seems to be influenced by both a higher willingness to report incidents by skydivers, and the better functioning of a safety manager, introduced at dropzones in 2011 [14]. Furthermore, the start of the COVID pandemic in late 2019 contributed to fewer jumps [15]. In several incidents, a link exists to the low number of jumps made in the last 12 months (currency). Currency is determined by the number of jumps made in the past 12 months. In general, the more aggressive the parachute characteristics the more jumps must be made to remain current. If a jumper (regardless the license) has not jumped for six months or more, he is not longer considered current [13].

Other studies have suggested that the increased popularity of high-performance canopies and aggressive flying techniques is a more significant contributing factor [1, 3, 14, 16]. Their findings agree with the finding in this study that 31% of the 234 injuries that occurred during landing were caused by an incorrect flare (parachute decelerations). This is a phase of the jump that is pushed to the limit in skydivers flying with high performance canopies and with aggressive flying techniques.

Combining these arguments (lack of currency and the increased popularity of high-performance canopies) leads us to an alternative explanation for the increased incidence as of 2016: the increasing popularity of wind-tunnel flying. In a wind-tunnel, freefall skills can be accelerated rapidly: 1 h of tunnel flying, which can be done in a day, compares to 60 jumps from a plane. Therefore, for people with relatively few skydives, the rapidly increased frefall skills could lead to an overestimation of canopy flying skills and consequently an increased risk of accidents [17]. Jumpers and their peers can overestimate their skills.

### Injury rate

The injury rate in this study is lower than the incidence of injury reported by previous (older) studies [3, 4, 18–20]. This difference could be explained by improvements in equipment (e.g., the change from round to square parachutes, and the implementation of automatic opening devices), better training (e.g., the implementation of accelerated freefall instructors), and a better understanding of contributing factors [7]. Furthermore, in 2011 the KNVvL decided to increase safety within the organization by introducing a safety management system [21].

### Comparison with other (extreme) sports

In skydiving, BASE jumping and paragliding, the incidence of injuries is expressed per jump. In other (extreme) sports, the incidence of injuries is expressed per 1,000 h of sports practice. To compare skydiving with other sports, we calculated the number of injuries per 1,000 h of sport practice, assuming that one jump is equivalent to 1 h of sport practice. This assessment was based on the following numbers; a preparation and parachute packing time of 15 min, a flight time of up to 20–30 min and a free fall or parachute flight time of 5–10 min. In this study, the incidence of injuries per 1,000 h of skydiving is 0.72. Skydiving compares favorably with other extreme sports, such as kitesurfing, mountain biking, and rock climbing, with 10.1, 16.8 and 9.8 injuries per 1000 h of practicing the sport, respectively [22, 23]. Regarding similar sports (e.g., BASE jumping and paragliding, with 393 and 1,080 injuries per 100,000 jumps, respectively), skydiving also compares favourably with 72 injuries per 100,000 jumps [24, 25].

### Skydiving injuries

From 1995 to 2020, the most common locations for an injury were the ankles/feet, lower legs and spine. The most common types of injury where fractures, contusions, and strains. This finding agrees with other studies that have also indicated a predominance of these types and locations of injury [1, 3, 10, 11, 26]. Most injuries occur during the landing phase in either the inexperienced and perhaps surprisingly, in the moderately to highly experienced jumpers.

The subanalysis combining technical and medical data for all skydiving incidents with an injury from 2015 to 2020 at Teuge indicated that most of the skydivers with minor injuries were inexperienced. Fractures of the lower legs and ankles are the most common injuries in that category, with a corresponding ISS of 4. The observation that inexperienced jumpers are more prone to accidents seems intuitive, as they are not yet fully skilled. Most likely due to the more docile canopies, their injuries are relatively minor. Within the group of skydivers with major injuries, about half were experienced to highly experienced (licence C/D), and the most common injuries were neurological damage to the head and spinal fractures. The increased and more major injuries of experienced jumpers may be explained by their use of aggressive and high-speed landing techniques, which became popular in the early 2000s. An increase in incidents for this reason has been observed previously [27, 28]. As discussed previously in the rise in incidences section, we believe lack of currency is an important contributor to errors of judgment during high speed landings.

### Prehospital and hospital care of the injured

In this study, outpatient treatment was insufficient for all of the included patients. All patients went to a Level 1 or 2 trauma center, and of those, 30% required an ambulance and 13.3% a trauma helicopter to reach the hospital. Ambulance and HEMS crews arriving at the scene of a skydiving accident should always assume that the person has fallen from a height (unless otherwise stated by the staff) and stabilize the patient according to the Advanced Trauma Life Support (ATLS) principles.

Experienced jumpers presenting to the emergency room after an accident should always be considered to have “high-energy trauma,” as depicted in serious injuries found in skydiving injuries. Treatment should follow the standard trauma guidelines according to the ATLS. Clinicians must be aware of spinal fractures and traumatic brain injuries and adequately assess the extremities to optimize the care and outcome of injured skydivers.

Clinicians should also be alert for a relatively rare injury in skydiving. The speed at which the parachute deploys and jumpers hit the ground can lead to friction between the skin and other materials (e.g., clothing, harnesses, lines and the parachute), which can lead to friction burns, a phenomenon already known to occur in road traffic accidents [29]. Previously, Ellitsgaard et al. also described these “friction burns” of injured skydivers [4].

### Protective measures

Most injuries occur during the landing phase. The vast majority injured skydivers were wearing low sport shoes without any rigidity at the ankle. Twelve skydivers dit not even wear a helmet. In the basic safety regulations for sports parachuting, the KNVvL stated that solid footwear is compulsory only for skydivers without a C license. A hard helmet is compulsory when a skydiver does not possess an A license, and head protection is compulsory when skydivers do not possess a C license [13].

As suggested by Ellitsgaard et al. in 1987, solid boots can protect the ankles during landing [4]. More recent studies have also demonstrated the effectiveness of ankle braces in preventing injuries [30, 31]. Westman et al. described the relatively low number of head injuries as an encouraging outcome of helmet requirements [11]. Therefore, the mandate of wearing solid shoes/braces and helmets for all skydivers could help to prevent injuries.

### Meteorological-related factors in skydiving

Previous studies have mentioned that knowledge gaps exist in the field of meteorological factors contributing to injuries in skydiving [1]. Weather-related factors, such as ground windspeed (at landing), wind speed at the opening height (is the intended landing area reachable?), and visibility or cloud cover are significant in skydiving. Rules are defined in the basic safety regulations for sports parachuting promulgated by the KNVvL [13].

In this study, most injuries occurred during conditions of good visibility and cloudy conditions with a ground wind speed of less than 5 m/s. In low visibility conditions or with high cloud coverage, skydiving is not permitted. Typically, the parachutist slows when landing against the wind. In low wind speed conditions, the parachutist can reach a *higher* speed at landing relative to the ground, which can be a critical contributing factor. This finding indicates that excessive speed at landing is a contributing factor to injury; however, it has not been previously reported in the literature [7]. Moreover, a comparatively higher percentage of severe injuries occur in skydivers landing with a higher ground wind speed.

### Strengths and limitations

This retrospective study is based on three databases covering a period of 25 years; with missing data and an unequal sample size, making statistics difficult. Therefore the relationships may only be suggested, except for the jump type variable. The database was completed by comparing skydiving injuries with hospital data (only for the 2015 to 2020 data). Furthermore, insufficient medical knowledge among skydivers can lead to misclassification bias in the database.

A further limitation is the relatively few cases included from the medical databases. Still, we believe this crucial data contributed to a better understanding of the injury patterns compared to what the technical database alone could provide. We believe the sample is representative of the whole group of injuries that occurred at the dropzone at Teuge. Because 39% of all the jumps were performed at Teuge, it is representative of all skydives performed in the Netherlands.

## Conclusion

Skydiving is not without risk. Starting from 2016, incidents have been increasing. Clinicians must be aware of spinal fractures and traumatic brain injuries and adequately assess the extremities/skin of the skydiver. Better skydiving registration and regulations are essential, focusing on prevention with stricter safety regulations (e.g., solid footwear, ankle braces, helmets and safer jump conditions) and increased safety awareness among jumpers (e.g., addressing peer pressure).

### Electronic supplementary material

Below is the link to the electronic supplementary material.

Supplementary Material 1

Supplementary Material 2

## Data Availability

The datasets used and/or analysed during the current study are available from the corresponding author on reasonable request.

## Abbreviations

ATLS: Advanced trauma life support
BASE: Building, antenna, span, and earth jumping
EHRs: Electronic health records
FAI: Fédération Aéronautique Internationale
HEMS: Helicopter Emergency Medical Service
ISS: Injury Severity Score
KNVvL: Royal Netherlands Aeronautical Association

## References

[1] Barthel C, Halvachizadeh S, Gamble JG, Pape HC, Rauer T. Recreational skydiving-really that dangerous? A systematic review. Int J Environ Res Public Health. 2023;20(2). 10.3390/ijerph20021254 PMC9859333 36674008

[2] Koninklijke Nederlandse Vereniging voor Luchtvaart. Springtechnisch jaarverslag 2022. https://www.parachute.nl/safety.html. Accessed 10 Jan 2024.

[3] Barrows TH, Mills TJ, Kassing SD (2005). The epidemiology of skydiving injuries: World freefall convention, 2000–2001. J Emerg Med.

[4] Ellitsgaard N (1987). Parachuting injuries: a study of 110,000 sports jumps. Br J Sports Med.

[5] Steinberg PJ (1988). Injuries to Dutch sport parachutists. Br J Sports Med.

[6] Lowdon IM, Wetherill MH (1989). Parachuting injuries during training descents. Injury.

[7] Fer C, Guiavarch M, Edouard P (2021). Epidemiology of skydiving-related deaths and injuries: a 10-years prospective study of 6.2 million jumps between 2010 and 2019 in France. J Sci Med Sport.

[8] The Fédération Aéronautique Internationale (FAI). Safety report 2009. https://www.fai.org/isc-documents. Accessed 08 Nov 2023.

[9] The Fédération Aéronautique Internationale (FAI). Safety report 2019. https://www.fai.org/isc-documents. Accessed 08 Nov 2023.

[10] Esser SM, Baima J, Hirschberg R (2013). Falling for sport: a case report of skydiving and SCI. Curr Sports Med Rep.

[11] Westman A, Bjornstig U (2007). Injuries in Swedish skydiving. Br J Sports Med.

[12] Ball VL, Sutton JA, Hull A, Sinnott BA (2014). Traumatic injury patterns associated with static line parachuting. Wilderness Environ Med.

[13] Koninklijke Nederlandse Vereniging voor Luchtvaart. Basis Veiligheidsreglement Sportparachutespringen 2022. https://airboss.nl/nl/download/. Accessed 21 Dec 2023.

[14] Koninklijke Nederlandse Vereniging voor Luchtvaart. Springtechnisch jaarverslag 2018. https://www.parachute.nl/safety.html. Accessed 28 Sep 2023.

[15] Koninklijke Nederlandse Vereniging voor Luchtvaart. Springtechnisch jaarverslag 2020. https://www.parachute.nl/safety.html. Accessed 28 Sep 2023.

[16] Koninklijke Nederlandse Vereniging voor Luchtvaart. Springtechnisch jaarverslag 2011. https://www.parachute.nl/safety.html. Accessed 28 Sep 2023.

[17] Kelsay CB. Development of Human Body Flight [Honors College Thesis]: Oregon State University; 2016.

[18] Amamilo SC, Samuel AW, Hesketh KT, Moynihan FJ (1987). A prospective study of parachute injuries in civilians. J Bone Joint Surg Br.

[19] Baiju DS, James LA (2003). Parachuting: a sport of chance and expense. Injury.

[20] Straiton N, Sterland J (1986). Sponsored parachute jumps–can they cause prolonged pain?. Br J Sports Med.

[21] Koninklijke Nederlandse Vereniging voor Luchtvaart. Handboek Veiligheid Management Systeem Afdeling Parachutespringen. https://www.knvvl.nl/parachutespringen/veiligheid/veiligheid-management. Accessed 21 Dec 2023.

[22] Bigdon SF, Hecht V, Fairhurst PG, Deml MC, Exadaktylos AK, Albers CE (2022). Injuries in alpine summer sports - types, frequency and prevention: a systematic review. BMC Sports Sci Med Rehabil.

[23] van Bergen CJ, Weber RI, Kraal T, Kerkhoffs GM, Haverkamp D (2020). Kitesurf injury trauma evaluation study: a prospective cohort study evaluating kitesurf injuries. World J Orthop.

[24] Soreide K, Ellingsen CL, Knutson V (2007). How dangerous is BASE jumping? An analysis of adverse events in 20,850 jumps from the Kjerag Massif, Norway. J Trauma.

[25] Feletti F, Aliverti A, Henjum M, Tarabini M, Brymer E (2017). Incidents and injuries in Foot-Launched Flying Extreme sports. Aerosp Med Hum Perform.

[26] Christey GR (2005). Serious parasport injuries in Auckland, New Zealand. Emerg Med Australas.

[27] Hart CL, Griffith JD (2003). Rise in landing-related skydiving fatalities. Percept Mot Skills.

[28] Vidovic M, Rugai N (2007). Are hook turns a major obstacle to safe skydiving? A study of skydiving fatalities in the United States from 1992 to 2005. Percept Mot Skills.

[29] Agrawal A, Raibagkar SC, Vora HJ (2008). Friction burns: epidemiology and prevention. Ann Burns Fire Disasters.

[30] Amoroso PJ, Ryan JB, Bickley B, Leitschuh P, Taylor DC, Jones BH (1998). Braced for impact: reducing military paratroopers’ ankle sprains using outside-the-boot braces. J Trauma.

[31] Schmidt MD, Sulsky SI, Amoroso PJ (2005). Effectiveness of an outside-the-boot ankle brace in reducing parachuting related ankle injuries. Inj Prev.

