# Supplementary material for: The ground is the limit: epidemiology of skydiving accidents over 25 years and in 2.1 million jumps in the Netherlands with sub-analysis of injuries reported by medical professionals in the past five years
Source: World J Emerg Surg. 2024 Feb 28;19:7. doi: 10.1186/s13017-024-00535-w (PMC10900578; doi:10.1186/s13017-024-00535-w)
Supplement: Supplementary file 1 — Supplementary Material 1 [file 13017_2024_535_MOESM1_ESM.docx]

**Additional file 1: Table showing demographic variables of jumpers who sustained injury in the period 1995 to 2020.**

| Jumper demographics of all injuries in the period 1995-2020 | Total nr. of incidents |  |  |  |  |  |  |
| --- | --- | --- | --- | --- | --- | --- | --- |
|  |  | student |  |  | experienced |  | unknown |
|  |  | AOR ^***^ | AOS ^***^ | AFF ^****^ | FF ^*****^ | Tandem |  |
| Skydiving incident resulting in injury. | 1503 | 25 | 136 | 42 | 174 | 54 | 1072 |
| Gender, n  Male  Female  Unknown | 1022  379  102 | 20  2  3 | 81  33  22 | 34  4  4 | 103  35  36 | 40  9  5 | 744  297  32 |
| Age, median  (range) | 26  (16-85) | 47  (30-65) | 28  (18-67) | 37  (18-66) | 37  (16-70) | 43  (20-62) | 30  (16-85) |
| Experience  Amount of jumps  <25  25-100  100-700  700-1200  >1200  Unknown  Currency  Amount of jumps^*^  <10  10-50  50-100  100-200  >200  Unknown | 182  27  45  25  62  1162  180  43  28  53  31  1168 | 15  1  4  0  1  4  15  3  1  2  0  4 | 106  0  1  1  0  28  108  1  0  0  1  26 | 31  1  0  0  5  4  31  1  0  2  3  4 | 17  24  40  22  24  47  13  38  25  39  4  55 | 13  1  0  2  32  6  13  0  2  10  23  6 | 0  0  0  0  0  1073  0  0  0  0  0  1073 |
| Canopy type ^**^  Cat I/II  Cat III/V  Cat VI/VIII  Unknown | 220  49  48  1186 | 1  1  0  23 | 107  1  0  28 | 29  3  3  6 | 70  44  12  48 | 13  0  33  8 | 0  0  0  1073 |
| Legenda. ^*^In last 12 months. ^**^see additional file 2^***^Automatic opening round/square. ^****^Accelerated free fall program. ^*****^Free fall. | | | | | | | |
