# Supplementary material for: The ground is the limit: epidemiology of skydiving accidents over 25 years and in 2.1 million jumps in the Netherlands with sub-analysis of injuries reported by medical professionals in the past five years
Source: World J Emerg Surg. 2024 Feb 28;19:7. doi: 10.1186/s13017-024-00535-w (PMC10900578; doi:10.1186/s13017-024-00535-w)
Supplement: Supplementary file 2 — Supplementary Material 2 [file 13017_2024_535_MOESM2_ESM.docx]

**Additional file 2: General information about Skydiving**

AOR stands for automatic opening with a round parachute (see picture B) and AOS stands for automatic opening with a square parachute (see picture C). A parachute opens automatically through a static line. A static line is a fixed cord attached to the aircraft and at the other end to the top of the jumper’s deployment bag (in which the canopy is packed). When a parachutist jumps from the aircraft, the static line becomes taut and then pulls the deployment bag out of the container. Next, the canopy can inflate as the jumper continues to fall. The difference between a round parachute and a square parachute is that a round drops straight down and relies solely on drag to slow your descent while the square is a wing that flies forward and therefore creates a certain amount of lift. The number of jumps with a round parachute decreased drastically and almost never occurs anymore.

AFF stands for accelerated freefall (see picture D) and is a method of skydiving training. Accelerated does not mean you fall downward faster: it means you can experience solo freefall, after only approximately 7 training jumps. The instructor holds on to the student until the student deploys their own canopy. Once the student has proven they can deploy their own canopy on the first few jumps, the student will be released on subsequent levels and will have the opportunity to prove to their instructors that they have the basic flying skills required to maneuver in freefall without assistance.

© Adobe stock

Tandem skydiving (see picture A) refers to a type of skydiving where a student skydiver is connected to an instructor by a harness. The instructor guides the student through the whole jump and the student needs only minimal instruction before making a jump.

Technically speaking, swooping (see picture E) is an advanced form of canopy flying where a person gains speed through specific maneuvers. while leaving enough time for the canopy to glide horizontally across the ground.

There are 4 types of skydiving licenses, categorized as A, B, C, and D. The A-license is the first license, and the D-license is the highest level of skydiving license. The A-license is proof that the skydiver has completed their training and is ready to jump without supervision. To obtain the A-license, a skydiver needs a minimum of 25 jumps and must demonstrate certain skills. A B-license is obtained when a skydiver has made 50 jumps, followed a course of precision landing and water landing, and showed some other maneuvers. The C-license is acquired when the skydiver made 200 jumps and the D-license when 500 jumps were made.
